# Variation in Community Structure and Abundance of Fish in Simple Structured Shallow Sandy Habitats

**DOI:** 10.1002/ece3.70381

**Published:** 2024-10-27

**Authors:** Lari Veneranta, Mats Westerbom

**Affiliations:** ^1^ Natural Resources Institute Finland (Luke) Vaasa Finland; ^2^ Natural Resources Institute Finland (Luke) Turku Finland

**Keywords:** Baltic Sea, beach seine, community structure, fish community, sandy beach, surf zone

## Abstract

Sandy beaches and their surf zones characterise many of the world's open coastlines. They are important breeding, nursery and feeding areas for many species of fish. Despite the commonness and importance of sandy beach surf zones, the dynamics, space occupancy and diversity patterns of residing fish is in many places poorly understood. The aim of this study was to (1) characterise the fish community structure in 11 simple structured sandy surf zones of the northern Baltic Sea and (2) relate variation in fish abundance and community structure to a set of chosen abiotic variables. Using beach seine, weekly or biweekly sampling was conducted at fixed sites at 10 occasions throughout a summer season. A total of 60,006 fish individuals belonging to 20 species were recorded. Changes in abundance and community structure were mainly driven by the variation of only five species reflecting species‐specific recruitment patterns and different spatial responses to abiotic variables. Dominating groups were Gasterosteidae, Ammodytidae and Gobiidae that together formed 86% of the total adult fish catches. Larval numbers were completely dominated by Gobiidae. Multivariate analyses indicated species‐specific responses to measured environmental variables, most important being a combination of wave exposure, beach slope, bottom roughness, and temperature. The present study shows that changes in fish abundance on simple structured sandy sublittoral beaches in the northern Baltic Sea are large over the course of a breeding season. It also reveals that variation in adult and juvenile fish are driven by a set of abiotic factors that influence on the fish assemblage structure through mainly species‐specific, rather than through generic responses. Unravelling the degree to which the sandy shore fish community vary in the northern Baltic Sea will help in managing coastal environments that are increasingly being threatened by many anthropogenic stressors.

## Graphical abstract

We characterised the fish community structure in simple structured sandy surf zones of the northern Baltic Sea and related variation in fish abundance and community structure to a set of chosen abiotic variables. Our results show that spatio‐temporal changes in total abundance and community structure of resident fish are mainly driven by the variation of a few dominating species.

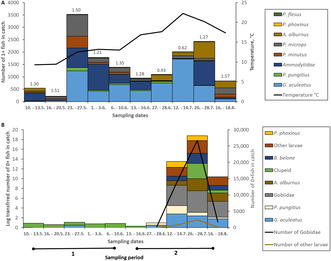

## 1 Introduction

Sublittoral sandy shores are dominant coastal habitats and important recruitment, nursery and feeding grounds for many fish (e.g., Gibson 1973; Luijendijk et al. 2018; Harris and Defeo 2022; Gold et al. 2023). Despite their structurally uniform appearance, sublittoral sandy shores are highly dynamic and variable environments (Rodil, Lastra, and Sanchez‐Mata 2006; Harris et al. 2014). They are the home to a well‐adapted community that can cope with the extreme hydrodynamic regimes that characterise these ecotopes (Barboza and Defeo 2015). The structure of communities on sandy shores is determined by the interplay of physical (such as heat, wave exposure and tides), chemical (such as salinity, turbidity and pH) and biological processes (such as competition, facilitation and predation) which magnitude vary in time and space (Rodil and Lastra 2022). Seasonal changes in the occurrence of species, including fish, are often determined by species specific behaviour patterns, including breeding, and feeding migrations of adults or seasonal settlings or migrations of young individual stages (Mariani 2001).

Fish form important components in the shallow sandy shore ecosystems in temperate regions, where they utilise sublittoral sandy shores as larval recruitment, nursery, and foraging areas (e.g., Gutiérrez‐Martínez et al. 2021). Typically, fish communities in the surf zone of sandy beaches are dominated by a few species that can occur in great numbers (Pessanha and Araújo 2003; Nakane, Suda, and Sano 2013; Olds et al. 2018). In the northern Baltic Sea, shallow sandy bottoms are essential habitats for many commercially unimportant small fish species. Because of their small market value, the population biology of these basal species has largely remained unstudied, despite the fact that their ecological value may be very high as many of them hold a key position in the nearshore food webs (Taal et al. 2017 with references). Understanding the drivers affecting the food base, including the structure of basal fish species, is important as sandy beaches are increasingly being threatened (Defeo et al. 2009). Sandy shores in the Baltic Sea, for example, are losing their oligotrophic character due to severe and ongoing eutrophication processes, whose impact is accelerating due to climate change (Veneranta, Hudd, and Vanhatalo 2013). Given the importance of sandy beaches as fish breeding, nursery and feeding areas, understanding the timing of habitat use by different fish species is important. Central to protecting and managing these habitats is a better understanding of the resident fish community structure and the factors that control these communities in time and space.

Spatiotemporal changes in the community composition of fish have generally been well studied, especially on both sides of the Atlantic Ocean and in the Mediterranean Sea (Abookire, Piat, and Robards 2000, Gordo and Cabral 2001, Franco et al. 2006, Gutiérrez‐Martínez et al. 2021). However, we still lack basic understanding on how fish select and use surf zone habitats and how environmental variables (such as water quality, wave climate, beach morphology) influence on the surf zone fish community (Olds et al. 2018). Most studies in the northern Baltic, have focused on seasonal abundance patterns of fish in complex vegetated habitats and how biotic and abiotic factors influence on the community composition (e.g., Thorman 1986; Nellbring 1988; Pihl et al. 1995; Vahteri, O'Brien, and Vuorinen 2009; Uspenskiy, Zhidkov, and Levin 2022), or more lately, studies have used modelling tools to predict the extent and location of reproduction areas (Florin, Sundblad, and Bergström 2009; Sundblad et al. 2009; Veneranta et al. 2011; Kallasvuo, Vanhatalo, and Veneranta 2017). While there are some data on temporal variations of fish assemblages of sandy littoral habitat in the Baltic Sea (Taal et al. 2017), it remains still virtually unexplored, how subtle changes in environmental characteristics at these simply structured sandy habitats—on relatively small geographical scale—are associated with the structure of local littoral fish communities. Consequently, we have much less comprehension of fish structure and dynamics in the shallow sandy shore habitat compared to many other Baltic Sea habitats. The data presented here will function as reference against which to detect a future change in the fish community structure in this specific system.

This paper addresses the following questions: (1) what is the daytime structure of the fish community in structurally simple environments over one summer breeding and feeding season? (2) Does the structure vary over time and space? (3) Which abiotic factors describe best the daytime fish community in simple structured sandy seascapes? Normally, researchers have selected shores over a span of varying structural habitat complexity to explain responses among fish, with often rather predictable outcomes. We selected shores that structurally were as close as possible, limiting the study to open sandy shore systems but that differed in abiotic terms (e.g., wave exposure, habitat size, temperature, slope). Our hypotheses were as follows: (1) wave exposure, bottom structure and habitat size affect fish community structure which (2) shows changes in structure over a summer season, largely following species‐specific migration patterns during breeding and changes in sea temperature. To test our hypotheses, we measured several abiotic parameters over a summer season and determined fish abundance and community structure for both adult and juvenile fish.

## 2 Materials and Methods

### 2.1 Sampling Area

**FIGURE 1:**
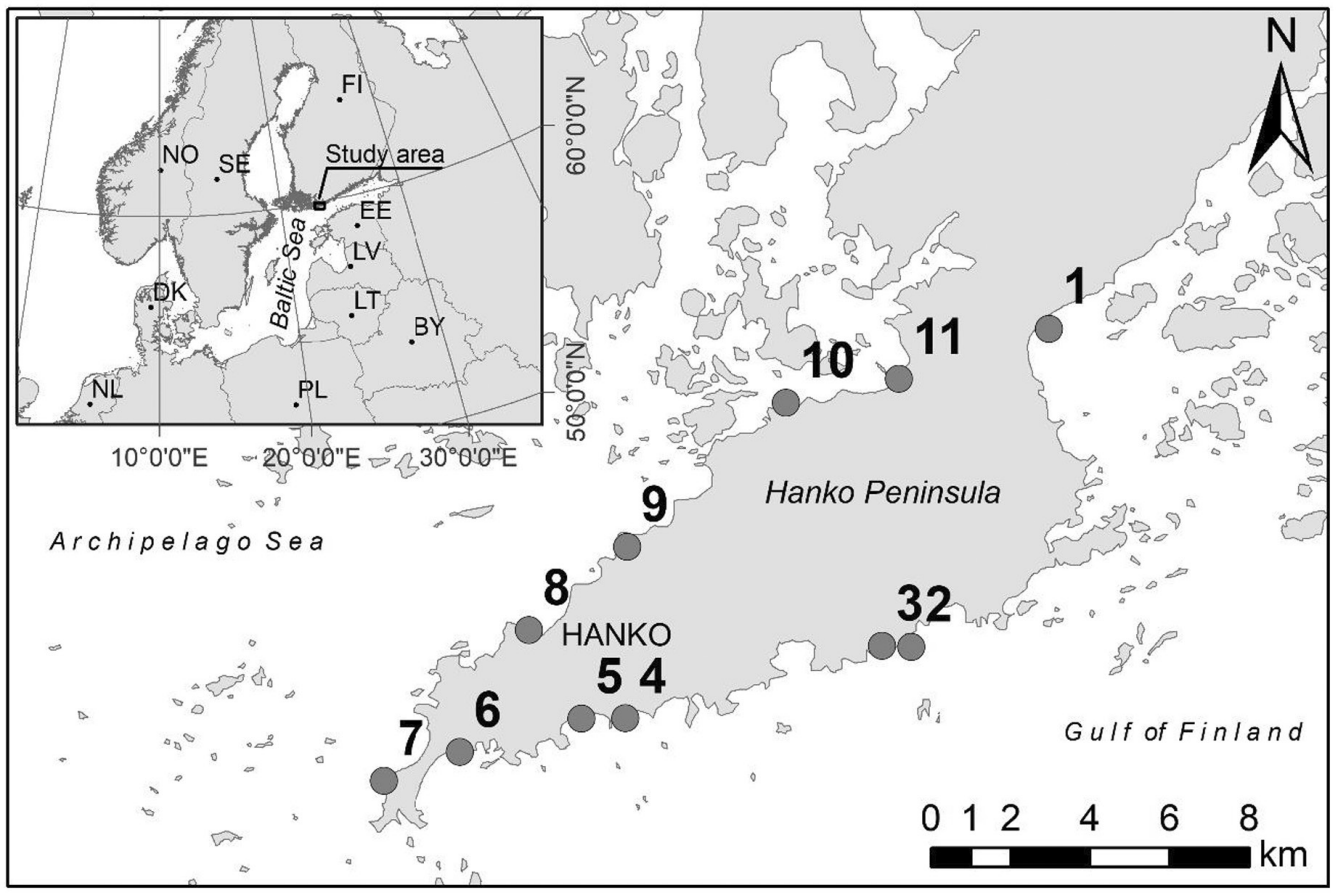
Research area on Hanko Peninsula. Numbers 1–11 represent the sampling sites.

The sampling was carried out from late spring to late summer at 11 sandy beaches dispersed on both sides of the Hanko Peninsula, northern Baltic Sea (Figure 1). The northern part of the study area is in a sheltered archipelago area on the north side of the Hanko Peninsula while the southern side is characterised by a narrow archipelago zone close to the open sea. At the Hanko Peninsula, sandy shores dominate the nearshore habitats, with alternating rocky shore outcrops that separates proximate sandy beaches. The salinity in the research area is low, varying between 5.5 and 7 PSU. In winter, the sea in south Finland is covered by ice for 2–4 months annually (Haapala et al. 2015). Sea temperature varies highly, both among and within seasons. In winter, the temperatures are close to 0°C and in summer months surface temperature may exceed 25°C. Upwelling in this area is frequent, causing water temperatures to drop dramatically during summer months (Haapala 1994). There are no tides, but periodic fluctuations in sea level occur due to changes in wind and air pressure.

### 2.2 Characteristics of Sampling Sites

Sampling sites varied in relation to wave exposure, steepness, extent of shallow bottom area, temperature and turbidity. The major bottom type was sand, with wave exposure‐dependent variation from silt to fine gravel. Vegetation in the study sites was scarce; only the two most northern sites (10 and 11) had low coverage of common reed (*Phragmites australis*) at the shoreside of the beach and perfoliate pondweed (*Potamogeton perfoliatus*) occurred deeper down (> 1 m). Low coverage of bladderwrack (*Fucus vesiculosus*) was found at some sites. Its total cover at the densest sites was estimated to be less than a few per cent.

### 2.3 Data Collection

Sampling was conducted on weekly basis from May 10 to June 13, 2005, and included five sampling occasions. This encompasses the spawning season of the main fish species. From June 16, sampling was conducted biweekly until August 18th, and included an additional five sampling occasions. To catch the fish, we used a beach seine, that had 8.5 m long and 1.4 m tall arms with a mesh size of 5 mm and a cod end with 1 mm netting. The width of the cod end was 1 m and length 3.7 m. The seine was set from the shore by walking and returning to shore (Leonardsson et al. 2016). The seine was set in a half‐circle by fording out from the shore and returning back with one end of the rope. Then, the seine was hauled back to shore. Each seine was trawled over a standard area of 125 m^2^. Three replicate trawls were done at each site at each sampling time at a maximum depth of ca. 1.3 m. The sampled shorelines were > 100 m long at all sites and the distance between replicate trawls were approx. 30 m. A total of 330 hauls were sampled, and each site was sampled at 10 occasions during the study. Upon trawling, fish were collected, identified, counted, and preserved in a 4% buffered formalin solution. In the laboratory, the fish were identified to species, calculated, and measured by length (TL) to the nearest 1 mm. The length distribution was used to separate young‐of‐the‐year (YOY) individuals from older fish. Early‐stage individuals of common goby (*Pomatochistus microps*) and sand goby (*Pomatochistus minutus*) are difficult to identify at the species level and given the extremely high number of these fish in the samples, the two species were treated as a single group. Also, juvenile herring (*Clupea harengus membras*) and juvenile European sprat (*Sprattus sprattus*) were grouped together to Clupeidae. Small sandeel (*Ammodytes tobianus*) and Great sandeel (*Hyperoplus lanceolatus*) were grouped to Ammodytidae and three species of flatfishes (*Platichthys flesus*/*Platichthys solemdali* and *Scopthalmus maximus*) were grouped as Pleuronectiformes.

At each sampling site, the main abiotic characteristics were collected, and the habitat types were identified (Appendix A). An area shallower than two metres was labelled ‘shallow’ and the extent of this area was defined using GIS. GPS was also used to calculate the average slope. The slope was estimated by walking perpendicular to the shoreline and measuring the angle from the waterline to the point where the water depth reached 2 m depth. Seabed coarseness was measured by measuring the average grain size of the bottom material. Grain size varied between 1 and 25 mm, and sites were grouped into four classes based on sand coarseness. Sparsely occurring stones in the hauling area were not considered. The presence of drifting algae and macrophytes was classified into two bivariate categories. Temperature, together with turbidity (Nephelometric Turbidity Units), was measured at every sampling. Also, automatic temperature loggers (Onset Hobo Water Temp Pro) measuring at 2‐h intervals were placed in the six sites (1, 2, 6, 8, 9 and 11) at a depth of 0.5 m. Exposure to wind and waves was measured using the Isaeus wave exposure index (Isæus and Rygg 2005).

### 2.4 Statistical Analyses

We used Principal Component Analysis (PCA) to condense and summarise the differentiation among the individual sites in relation to the seven environmental variables to broadly picture the sites in terms of environmental variables. For analysis, we used mean values for each environmental variable for each site and overlay vectors were used to visualise which of the environmental variables that best differentiated the sites. Prior to testing, environmental variables were fourth‐root transformed, normalised and checked for skewness or outliers.

Multivariate analysis within the PRIMER 7.0 package with the PERMANOVA+ add‐on was then used to investigate whether the adult fish assemblage structure varies among sites (11 levels), summer period (2 levels) and sampling dates (10 levels) nested within summer period. Community analyses were restricted to adult fish as young‐of‐the‐year (YOY) fish were completely dominated by Gobidae. The sum of three replicate hauls per site per sampling time was used in analyses. Prior to testing, data were fourth‐root transformed to reduce the influence of highly abundant species and thereafter checked for group homogeneity (PERMDISP). A dummy variable of one was added to the data to cope with completely empty samples. PERMANOVA calculates Pseudo‐F from a distance/dissimilarity matrix and discriminates group differences. When significant differences were observed (*p* < 0.05) among groups, pairwise comparisons were run. Univariate PERMANOVA was done on matrices based on Euclidean distances to check for monthly differences in the overall abundance of fish.

Furthermore, to explore the relationship between the structure of the fish community and the measured environmental variables, we used a non‐parametric distance‐based linear model (DistLM) on forth‐root transformed data. As environmental variables had different measurement scales, the data was normalised prior to testing. *R* ^2^ was calculated for each explanatory variable, and the most parsimonious model was selected according to the Akaike Information Criterion (AIC). The BEST procedure was used to examine the value of the selection criterion for all possible combinations of environmental variables that best explained the community (Anderson, Gorley, and Clarke 2008). We visualised the final model by distance‐based redundancy analysis, dbRDA, which is an ordination technique constrained to find linear combinations of predictor variables that explain the greatest variation in the data cloud (Anderson, Gorley, and Clarke 2008). Prior to all multivariate tests, Draftman plots were used to evaluate for multi‐collinearity and skewness of data. As collinearity was not an issue, all variables were included in the model.

Variation in the abundance of the most common individual fish species was analysed using General linear modelling (GLM) tools. GLM allows a more versatile analysis of correlation than standard regression methods because the error distribution of the dependent variable and the function linking predictors to it can be adjusted to the characteristics of the data (SAS OnlineDoc: Version 8). A General Estimating Equations (GEE) procedure of SAS statistics (v. 9.1.3) was used in analyses since it allows variables to be correlated (Liang and Zeger 1986). Fetch, shallow water area and steepness were partially interrelated factors in the GEE‐model, but r values were in all combinations < 0.7. All biological and physical data were tested for normality with normal probability plots and Kolmogorov–Smirnov tests. Steepness of the shoreline, wave exposure, shallow water surface area, bottom coarseness, temperature and turbidity were included in the analysis as environmental data. For analyses, a binomial distributed error and logit link were used to model the quantity of fish species at the shoreline and at the microhabitat scale. Differences between sampling sites in the density and presence of fish species were controlled by incorporating site as a repeated subject in the analyses. Over or under‐dispersion were evaluated with value of Pearson χ^2^/df.

## 3 Results

### 3.1 Abiotic Factors at Sampling Area

**FIGURE 2:**
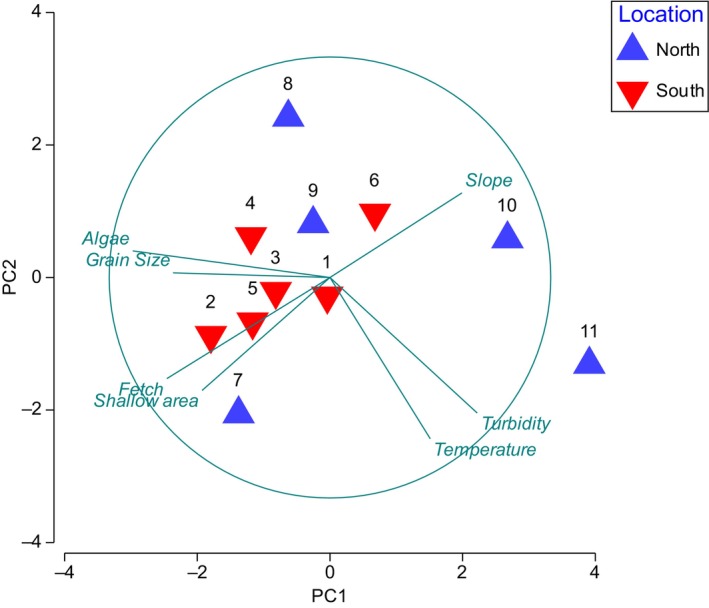
Principal Component Analysis (PCA) summarising sites based on normalised environmental variables. Southern sites (number above symbol – see Figure 1) are more clustered (homogeneous) than northern sites.

Principal Component Analysis (PCA) indicated some degree of environmental variability among the 11 sites. Sites 7, 10, 11 and to some extent 8, were separated from the remaining sites (Figure 2). PC1 (45.6%) and PC2 (22.1%) explained together 67.7% of the variation in the environmental data cloud. PC1 discriminated sites mainly based on Fetch (Wave exposure) with sites 7, 5, 4 and 2 showing highest wave exposure and sites 10 and 11 the least. Also, seabed coarseness varied mainly along PC1 with PC2 discriminating sites mainly based on temperature with sites 11 and 7 showing the highest temperature and sites 8 and 9 showing the lowest.

### 3.2 Species Composition

**FIGURE 3:**
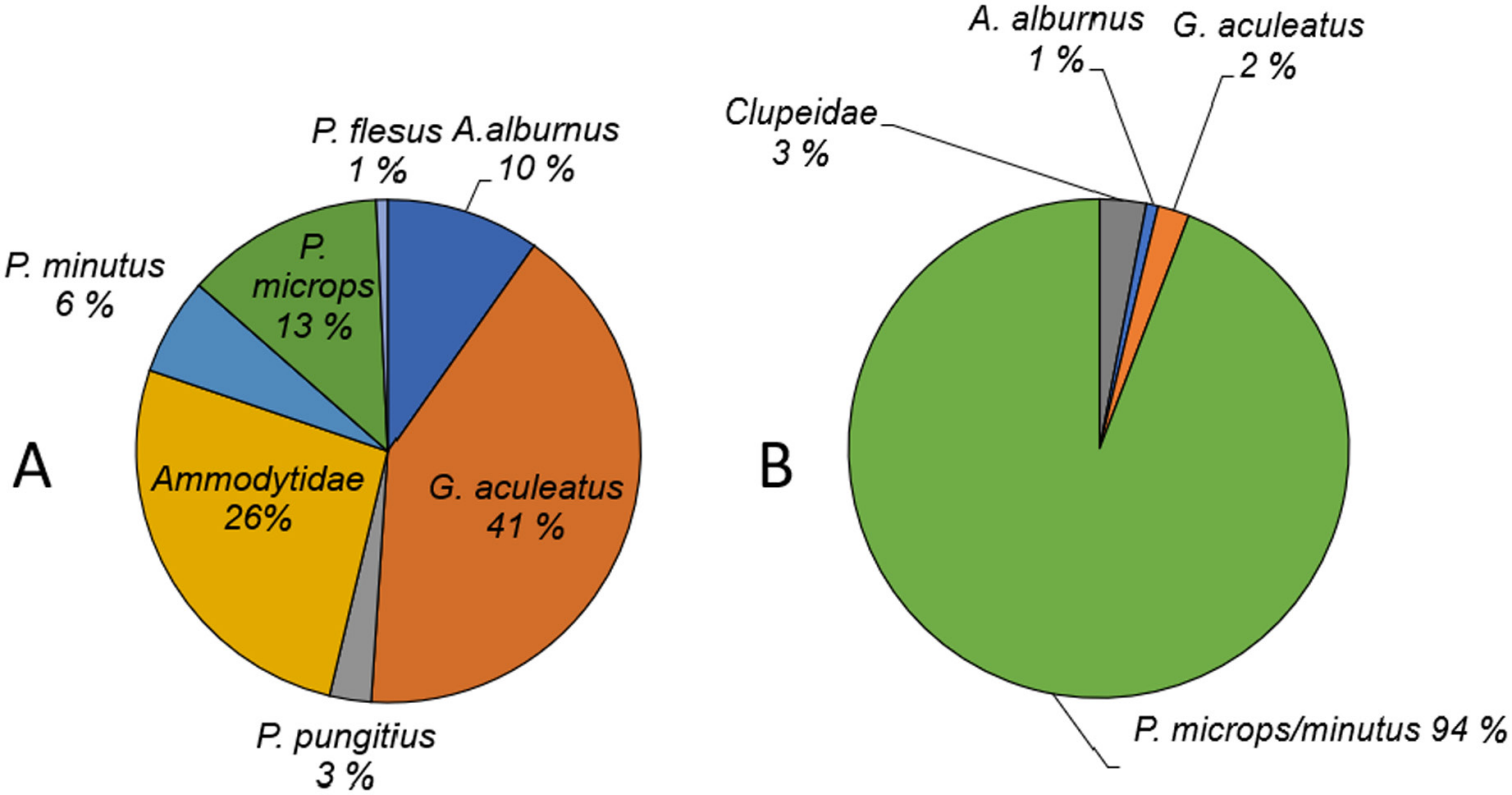
The total catch percentage of most common species for (A) ≥ 1+ and (B) 0+ fishes. *Pomatochistus microps* and *P. minutus* larvae were not identified as species and thus are combined for 0+ fishes.

**FIGURE 4:**
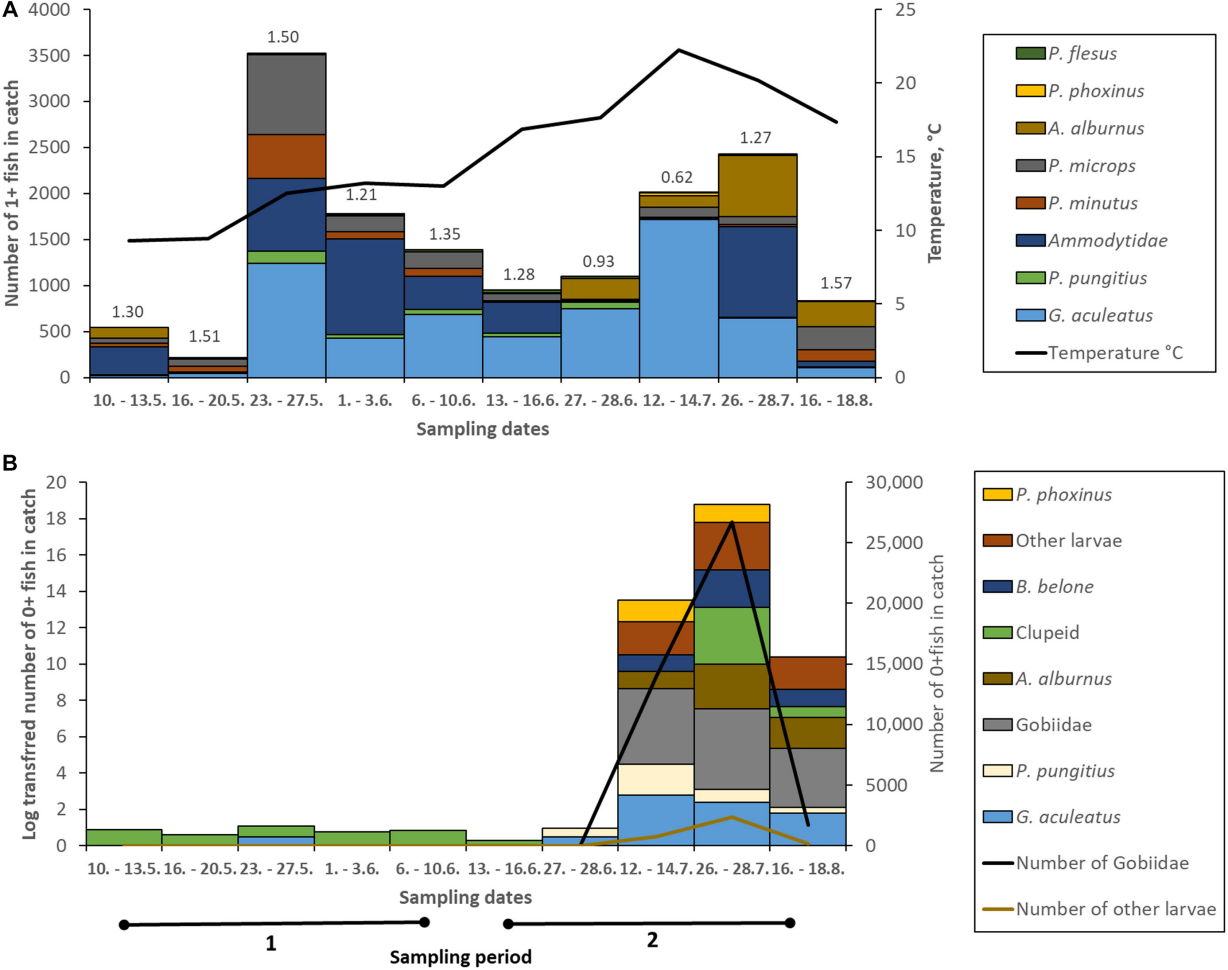
Temporal change in the fish community for 1+ or older fish (A) and juveniles (B). The numbers above bars in graph A indicate the Shannon‐Wiewer diversity index. Temperature (line graph in A is the average temperature over all sampling sites. Sampling rounds were divided into two periods. In graph B, both the log‐transformed number of larvae (bar graph) and an untransformed number of larvae (line graph) are shown).

A total of 60,006 individuals that belong to 20 fish species were recorded at the 11 sites on both sides of the Hanko Peninsula (Appendixes A and B). The average catch per site was 327 ± 1175 (SD) individuals and 4.1 ± 1.8 (SD) species. Dominating taxa were *Gasteroscus aculeatus*, Ammodytidae, *Pomatochistus microps*, *Alburnus alburnus* and *Pomatochistus minutus*. These species accounted together for over 95% of the total catch of adult fish. Gobies (*P. minutus* and *P. microps*) formed 94% of the fish larvae (Figure 3). Species present in all sampling periods were *G. aculeatus*, *P. pungitius*, *P. minutus* and *P. microps*. *G. aculeatus*, *P. minutus*, *P. microps* and Ammodytidae were found at all sites. *P. pungitius*, *B. belone*, *P. flesus*, Clupeidae were absent only at a few sites. Only on a few occasions, were other species abundant (Figure 4). The total number of fishes was highest in July due to the presence of fish larvae (578.7 ind/m^2^), whereas it was lowest in June (31.7 ind/m^2^) before the main fish breeding season (Univariate PERMANOVA pseudo‐*F* 7.1, *p* < 0.001). PERMANOVA also showed that there were no differences in species richness among the sites (pseudo‐*F* 0.9, *p* = 0.52).

On shallow shores, the first larvae were found in May and included Clupeidae, three spined sticklebacks (*Gasterosteus aculeatus*) and eelpout (*Zoarces viviparus*). In June, larvae consisted of a few whitefish (*Coregonus lavaretus*) and sticklebacks. Most larvae were found in July and August and the total number of larvae species was 11, including also several garfish (*Belone belone*) larvae (Appendix B). The catch of 0+ fishes consisted almost completely of Gobiidae. The share of other species was very low (Figure 4).

Gasterosteidae were also frequent, as well as Ammodytidae. A low number of YOY Clupeidae was present in early spring, and highest numbers were found in late July. The presence of YOY fishes increased sharply in mid July. In mid‐July, the number of 1+ fishes increased, mostly due to the high number of *Gasterosteus aculeatus*.

### 3.3 Community Structure and Abiotic Factors

The PERMANOVA analysis revealed a significant difference in the adult fish community composition across sites (pseudo‐*F* 5.8, *p* < 0.0001), sampling period (pseudo‐*F* 3.9, *p* = 0.015) and sampling dates nested within sampling period (pseudo‐*F* 2.6, *p* < 0.0001). Pairwise comparisons revealed that: (a) sites 7, 10 and 11 differed the most from the remaining sites (Table 1) (b) there was higher similarity among sites in the community composition early in the season (period 1) compared to the mid and late summer season (period 2). Marginal tests in DistLM identified seven potential environmental variables that each explained a small portion of the variation in the fish community structure. Among the significant explanatory variables, fetch (9%, *p* < 0.001), bottom coarseness (7%, *p* < 0.001) and temperature (6%, *p* < 0.001) were the main factors determining the fish community composition when tested alone without the influence of other variables. The overall BEST solution (*R* ^2^ = 0.21) based on AIC selection criterion was found by including fetch, slope, shallow area, bottom coarseness and temperature, whereas turbidity and occurrence of drifting algae were not included in the final model.

**TABLE 1:** Average similarity between and within sites over the course of the study.

Average similarity between/within groups												
**Site**	**1**	**2**	**3**	**4**	**5**	**6**	**7**	**8**	**9**	**10**	**11**	**Median**
**1**	50.7	38.1	40.9	39.1	40.8	45.1	31.9	38.8	44.5	39.7	28.4	39.4
**2**	38.1	56.5	49.8	55.2	51	51	47.9	54.7	48.2	42.1	42.7	49
**3**	40.9	49.8	45.5	46.2	49.5	45.2	39	46.5	46.7	41	38.4	45.7
**4**	39.1	55.2	46.2	61.4	49.8	52.1	45.4	48.3	50	33.2	30.4	47.3
**5**	40.8	51	49.5	49.8	48.4	48.6	40.5	47.4	47.4	38	35.6	47.4
**6**	45.1	51	45.2	52.1	48.6	49.4	41.6	47.2	48.5	38.6	29.3	46.2
**7**	31.9	47.9	39	45.4	40.5	41.6	55.1	48.9	37.5	31.7	33.8	39.8
**8**	38.8	54.7	46.5	48.3	47.4	47.2	48.9	52	44.1	42.5	42.9	46.9
**9**	44.5	48.2	46.7	50	47.4	48.5	37.5	44.1	46	37.5	30.3	45.6
**10**	39.7	42.1	41	33.2	38	38.6	31.7	42.5	37.5	49.5	49.6	39.3
**11**	28.4	42.7	38.4	30.4	35.6	29.3	33.8	42.9	30.3	49.6	60.2	34.7
**Median**	39.4	49	45.7	47.3	47.4	46.2	39.8	46.9	45.6	39.3	34.7	50.7

*Note:* Significant differences among groups are indicated with green colour, dark *p* < 0.001, median *p* < 0.01 and light green *p* < 0.05. Sites on the southern side of the Hanko peninsula are labelled yellow, and those on the northern side are labelled blue.

**FIGURE 5:**
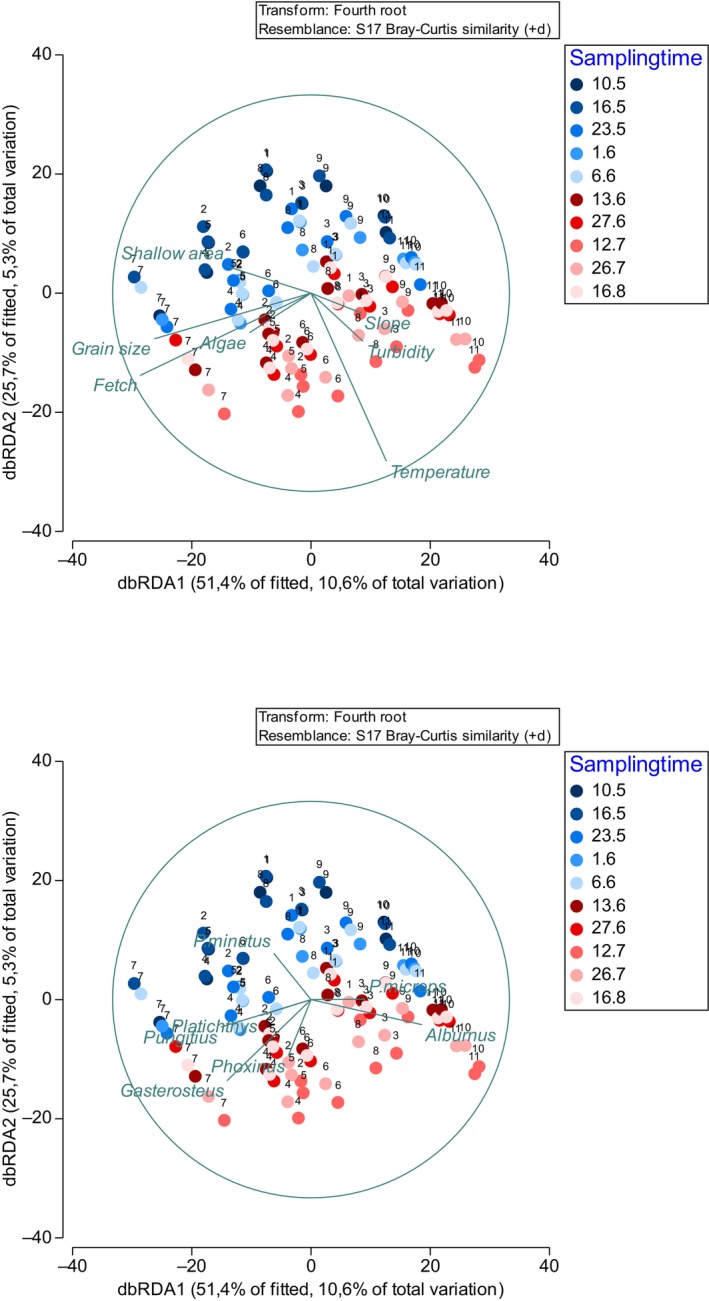
Distance‐based redundancy analysis (dbRDA) on fourth‐root‐transformed species abundance data with the most important discriminating environmental/background variables and species as vector overlays. Time of sampling shown with symbols, while numbers indicate the site (see Figure 1).

The high similarity among sites was mainly driven by Gasterosteidae and Gobiidae. Except for Cyprinidae, species of the same taxa were found at sites with similar abiotic environments. dbRDA plots indicated that, species could broadly be classified into three groups: (1) Gasterosteidae and Pleuronectidae, found in exposed and coarse bottomed sites, (2) *P. microps* and *A. albula* that were found at sheltered sites, and (3) *P. minutus* whose occurrence were driven mostly by temperature (seasonal changes; Figure 5).

The GEE–model indicated that significant factors affecting to occurrence of *G. aculeatus* were temperature and bottom coarseness (Table 2). *P. pungitius* was found at sites with higher temperatures. For the occurrence of Ammodytidae and Pleuronectiformes the steepness, shallow water surface area and exposure were statistically significant factors, and the largest samples were found from sites with a gentle slope, high exposure, and a large surrounding shallow water area. *P. minutus* favoured study sites with a gentle slope, large shallow water area, high exposure, cool and clear water as well as more coarse sand bottoms. *P. microps* seemed not to favour any measured abiotic factor. This species was found at all sites around the Hanko Peninsula, though the majority of ≥ 1+ individuals were caught from the northern side with higher turbidity and higher silt on bottoms.

**TABLE 2:** Generalised estimating equations model results of abiotic factors affecting fish occurrence for both, 1 year old and older fishes and young of the year fishes.

Species or taxa	Abiotic factor						
	Steepness^a^	Shallow water area^b^	Exposure to wind^c^	Temperature^d^	Bottom coarceness^e^	Turbidity^f^	Pearson χ^2^/df
≥ 1 year old fishes							
*G. aculeatus*	−0.111	−0.021	0.921	0.120*	0.752*	−0.126	1.111
*P. pungitius*	−0.491	−0.029	1.135	−0.151*	0.251	0.291	1.406
*Ammodytidae*	−2.657**	−0.188**	3.598**	−0.220**	−0.526	−0.084	1.303
Platichthys. sp	−2.390**	−0.186**	2.431**	−0.088	−0.509	−0.073	0.910
*P. minutus*	1.438**	0.098*	−1.426*	−0.184**	0.936**	0.388*	0.985
*P. microps*	0.998	0.087	−1.558	0.038	−0.080	0.395	0.855
*A. alburnus*	7.4198**	0.528**	−14.981**	0.297**	−2.372*	0.006	0.409
*P. phoxinus*	3.174**	−0.034	2.742*	0.460**	0.089	−1.687**	0.223
Young of the year fish							
*G. aculeatus*	−0.338	−0.001	0.0295	0.792**	0.189	−0.685*	1.062
*P. minutus/microps*	−0.833	−0.031	−0.255	1.241**	−0.361	−0.697**	0.553
*Clupeidae*	−2.388**	−0.159*	2.251	0.407**	0.788	0.183	0.755
*B. belone*	−0.010	0.096*	−1.735*	0.767**	0.777*	**−0.016**	0.475
*A. alburnus*	3.341**	0.249**	−6.283**	1.127**	−0.029	−0.709*	0.308

*Note:* Statistically significant values are bolded, *p < 0.05 and **p < 0.01.

^a^ (+) steeper/(−) gentle slope.

^b^ (+) lower/(−) wider shallow water surface area.

^c^ (+) more exposed/(−) less exposed to wind.

^d^ (+) higher/(−) lower temperature.

^e^ (+) more coarse/(−) less coarse bottom.

^f^ (+) clear water/(−) turbid water.

Since most of the fish larvae appeared in July (Figure 4B), the dominant factor for the larvae seems to be temperature. Especially for the clupeid larvae, also gentle slope appeared to be important. *A. Alburnus* on the other hand, favoured steeper beaches with lower wave exposure. Except for clupeid and *Belone belone*, water turbidity did not affect the occurrence of fish larvae (Table 2). Larvae of the fish clustered in a similar way as the 1+ fish. Gasterosteidae and *P. phoxinus* were found together. Clupeidae were predominately found in the shallow areas of the southern side of the Hanko Peninsula. Also, larvae of *Belone belone* were found mainly on the southern side together with larvae of Gobiidae. YOY *A. alburnus* was found almost exclusively on shallow and fine‐grained sites of the northern side of Hanko Peninsula.

## 4 Discussion

The fish assemblage structure on sublittoral sandy beaches varies in time and space reflecting changes in environmental parameters which are displayed as changes in functional or life history attributes of species (Poff and Allan 1995; Angermeier and Winston 1999). Although descriptive studies on the community structure of shallow water fishes are numerous, in the northern Baltic Sea studies on the temporal variation in the species composition and the abundance patterns of fishes in sandy surf zones has remained largely unstudied (see, e.g., Taal et al. 2017). This study demonstrates that (1) the spatio‐temporal changes in total abundance and community structure of resident fish were mainly driven by the variation of a few dominating species (Figure 3) and (2) the explanatory power of the measured independent factors was low for the community as a whole but it explained some abundance variations among distinct species. Results are in line with several past studies suggesting that the structure of surf zone fish assemblages varies over the progression of the summer season where physical attributes may explain little of the species composition (see, e.g., Inoue, Suda, and Sano 2008, with references). Overall, this study corroborates earlier research, suggesting that shallow sandy beach surf zones are characterised by a high numerical dominance of a few fish species, where a few families can be extremely abundant (Olds et al. 2018).

The abundance of species and the species composition in the studied area were similar to the few previously published studies in the northern Baltic Sea where the number of fish species vary between 15 and 31 species (Appendix C). Environmental variables explained some of the overall community structure (Figure 5) and abundance of single species, but the explanatory power was relatively low (see also, e.g., Rishworth, Strydom, and Potts 2015, Franco et al. 2016). In accordance with the hypotheses, wave exposure, beach slope, beach area, bottom coarseness and temperature were the most influential factors. High wave energy beaches are often characterised by a decrease in fish diversity and abundance (e.g., Inui et al. 2010; Shah Esmaeili et al. 2022). Expectedly, Ammodytidae and Pleuronectiformes reacted positively to increasing wave exposure, whereas *A. Alburnus* were more common at sheltered sites. Ammodytidae and Pleuronectiformes which are highly adapted to the beach environment can take the advantage of sand bottom as a hide and nutrition resource. In line with several past studies, the present study also indicated a notable association between beach slope, beach area and bottom roughness on species composition (e.g., Nakane, Suda, and Sano 2013). Structurally complex habitats in sandy environments, characterised by higher bottom roughness, or drifting macroalgae, may affect fish community composition through changes in shelter and food availability, but also through enriching the habitat for larvae, promoting larval settlement (de Souza, dos Santos, and dos Santos 2018). For example, Crawley et al. (2009) showed higher abundances of fish larvae and juveniles in the sandy surf zone when drifting macroalgae were present compared to sites where drifting algae were absent. Sundell (1994) found that the number of sticklebacks and gobies was higher in vegetated sites compared to structurally simple sandy beaches. In this study, coarse bottoms and high exposure were factors that appeared to interact with high quantities of *Gasterosteus aculeatus* whereas numbers were lower at simple structured sites. *G. aculeatus* numbers also varied in time. Previous studies (Bobsien 2006; Bergström et al. 2015) have shown that *G. aculeatus* mainly occur in the open sea area except during their breeding time. Here, the number of *G. aculeatus* were relatively low or absent in early and mid‐May, to increase and even become dominant at some sites from late May onwards, especially in places where habitat architecture was more complex. This observation fits well with the timing of breeding and migrating of *G. aculeatus* when they move from their wintering areas at open sea to their shoreward breeding areas.

Numerous studies have shown that temperature and salinity are the main factors affecting the structure of littoral fish assemblages in temperate regions (Hoff and Ibara 1977; Maes et al. 1998; Harris, Cyrus, and Beckley 2001). We did not measure salinity, but effects of temperature (which may be a proxy for ontogeny and reproduction timing) was clear (Figure 4). The low abundance of fishes in early spring may be due to their short life cycle and high winter mortality, especially among gobies that dominated the fish fauna. Many also move deeper down during winter and return to the shallows to breed in early summer. Most of the gobies in SW Finland are one‐ or two‐year‐old, and the winter mortality has been estimated to be as high as 80%–90% (Oulasvirta 1988). Nellbring (1986) found that breeding gobies arrive in early May at water temperatures of 7°C or less. In our system, most of the gobies arrived at the shallows only after the temperatures had risen to above 10°C. Nellbring (1986) argued that spawning time and site preferences of *Pomatochistus minutus* and *Pomatochistus microps* differ as *P. minutus* favour deeper water areas, coarser bottoms, and colder sites, whereas *P. microps* spawn later in shallows characterised by finer sand. We could not observe similar patterns as both *P. minutus* and *P. microps* were seen at the same sites, especially in May and June. Nonetheless, as seen in Figure 5, *P. microps* were drawn to sites with finer bottom material, whereas the catch of *P. minutus* was low when water temperature was high. Similar results were indicated by Hesthagen (1977) and Nellbring (1985) who suggested that *P. minutus* do not occur in shallow waters when water temperatures exceed 20°C. In this study, the number of 1+ and older gobies decreased in July when temperatures were at their highest. *P. microps*, on the other hand prefer higher temperatures than *P. minutus* (Fonds and Veldhuis 1973), and they were found abundantly also during temperature highs.

*A. Alburnus* and *Phoxinus phoxinus* were the only common species belonging to Cyprinidae in the studied habitats. In structurally more complex areas, Sundell (1994) and Lappalainen and Urho (2006) found a high abundance of YOY roach (*Rutilus rutilus*). The near lack Cyprinids in sandy exposed habitats of Hanko Peninsula, probably are explained by the low salinity tolerance of roe and juveniles and a preference for sheltered and more complex vegetated habitats for juvenile cyprinid recruitment (Härmä, Lappalainen, and Urho 2008). Also, the susceptibility of open shores to upwelling originated cooling can make these areas unsuitable for larvae of cyprinids or percids (e.g., Guma'a 1978). On other hand, adult roach are known to use areas outside sandy shores for feeding (Lappalainen, Westerbom, and Heikinheimo 2005) Therefore, *A. Alburnus* and *Phoxinus phoxinus* appears to be the only Cyprinidae that can take advantage of the harsh conditions in open shallow sandy areas of the northern Baltic Sea.

When interpreting results, it is noteworthy that associations found between community structure, fish abundance and environmental variables do not necessarily reflect causal relationships. Variables that we did not measure may have affected patterns seen. Such variables could be the influence of proximate neighbouring habitats (e.g., seagrass and macrophyte beds, rocky outcrops, reefs with macroalgae) where landscape connectivity processes could well explain the relatively low predictive power of the measured environmental variables, and the relatively high number of species occurring in low numbers. Franco et al. (2006) has suggested that sparsely vegetated and unvegetated sandy habitats probably act as ‘buffer’ and migration zones between more complex habitats. Fish move from surf zones to other habitats to feed and spawn (Olds et al. 2018; Mosman et al. 2020). For many species occurring infrequently and in low numbers in this study, sandy shores appeared to be transient habitats for fish, possibly migrating between other coastal habitats (Cowley, Whitfield, and Bell 2001; Vargas‐Fonseca et al. 2016). Most fish therefore seem to use northern Baltic Sea surf zones more as feeding and transit habitat, and only few species use it as spawning or juvenile nursery habitat. An exception is *Coregonus lavaretus* whose larvae is commonly observed in exposed sandy shores in the northernmost Gulf of Bothnia (Veneranta, Hudd, and Vanhatalo 2013).

There are some methodological limitations that need to be addressed. While beach seining is a common method for assessing the abundance of small and juvenile fish in littoral zones (e.g., Leonardsson et al. 2016; Hernández‐Álvarez et al. 2023; Jůza et al. 2024), it has some shortcomings. According to Lyons (1986), beach seine catch‐per‐effort does not accurately represent neither density nor the relative abundance of fish since all fish present in an area seined are not captured and certain species are clearly more vulnerable to capture than others. Fast swimming species may avoid the seine and very small juveniles may pass through the nets (e.g., Urho 1997). Paloumpis (1958) and Higer and Kolipinski (1967) have found that fish preferring shallow areas are easier to catch at nighttime. Taal et al. (2017), showed that some species in the shallow sandy beach habitat of the northern Baltic Sea show diel variation. We sampled shores only at daytime and only over one summer season, and the fish densities and species composition might be different at nighttime and during other times of the year. For practical reasons—considering the number of sites, number of replicates within sites and 10 sampling occasions—sampling was in this study standardised to occur only at daytime between 09.00 and 19.00 and only spanned one summer season. It is therefore important to bear in mind that some of the fishes in this study show strong diel behaviour (Taal et al. 2017) and probably also seasonal changes in distribution. Some species are night active, and hide in other habitats during the daylight hours, or are found deeper down, during the day, and possibly where not caught or were caught in low numbers only. For example, *P. minutus* swim more actively at darkness (Ehrenberg and Ejdung 2008). Vice versa, Ammodytidae stay buried at night and are active at daytime (Winslade 1974). While it is important to keep in mind these methodological issues, our intention was not to evaluate the capture efficiency of the seine, nor to evaluate diel variation, or changes over the year, but to use a standardised method to systematically evaluate the daytime composition of small fish occurring in the sandy surf zone during a summer season.

### 4.1 Exposed Sandy Beach as a Breeding and Juvenile Habitat

Typical for structurally simple sandy beach surf zones are a small number of species where few species dominate (Elliot and Dewailly 1995; Gordo and Cabral 2001; Franco et al. 2006; Olds et al. 2018). The result of this study indicates that simple structured sandy littoral zones function as a marginal breeding area for most of the species occurring in shallow littoral habitat of the northern Baltic Sea, but for some species they are highly important. Thus, our findings only partially agree with the common notion saying that sandy beaches are critical recruitment and nursery grounds for fish. Only 11 YOY species were recorded and Gobiidae larvae dominated almost completely the YOY community. Highly adapted species, like Gobiidae and Pleuronectidae can take advantage of the prevailing harsh conditions in shallow sandy habitats which are core areas for the population of these species in the sense of breeding and nursery (Pihl, Wennhage, and Nilsson 1994, Beyst, Hostens, and Mees 2001, with references). Temperature was the foremost factor explaining the occurrence of fish larvae, which strongly affects reproductive timing and larval maturity (e.g., Thorman 1986; Taal et al. 2017).

In summary, our results show that the sandy surf zone fish community in the northern Baltic Sea is structured by an interplay of many environmental variables and influenced by the progress of the summer season. The high abundance of a few species in the structurally simple sandy environment can be explained by the few dominant species taking advantage of littoral zones as larval nutrition and breeding area. These species have adapted to live in extreme conditions utilising the limited biotic and abiotic resources that characterise open simple structured sandy shores. However, for most of the species sampled in this study, simple structured sandy beaches most probably are through‐passage environments. In conclusion, more research is needed both to characterise sandy surf zone fish assemblages in the northern Baltic Sea and to better understand the causal mechanisms that structure these communities. Better understanding of the dynamics and spatial patterns of the fish community in the sandy surf zone is needed as the first step towards the development of effective measures to protect these habitats including the ecologically important basal species they host.

## Author Contributions

**Lari Veneranta:** conceptualization (equal), data curation (equal), formal analysis (equal), methodology (equal), validation (equal), visualization (equal), writing – original draft (equal), writing – review and editing (equal). **Mats Westerbom:** conceptualization (equal), data curation (equal), formal analysis (equal), methodology (equal), validation (equal), visualization (equal), writing – original draft (equal), writing – review and editing (equal).

## Conflicts of Interest

The authors declare no conflicts of interest.

## Supporting information


Data S1.

## Data Availability

All the required data are uploaded as supplementary material.

## Appendix A Abiotic Features Describing the Sampling Sites, Including Also Data on Fish Abundance and Species Number

**Table:**

Abiotic features					Fetch	Sum of turbidity (NTU)	Heat sum1 (°C)	Heat sum2 (d/°C)	Fish data	
Site	Steepness (%)	Coarseness (i)	Exposure (i)	Shallow area (ha)					Mean Individual abundance	Mean Number of species
1	0.9	3	2.4	41.2	13,319	4.2	62.7	1648	34 ± 13	2.8 ± 1.5
2	0.2	3	3.4	32.6	64,532	5.1	62.6	1782	1113 ± 2989	5.4 ± 1.6
3	0.2	2	1.3	19.9	22,512	5.4	65.9	—	27 ± 26	4.3 ± 1.7
4	1.5	3	3.2	5.9	65,507	5.9	62.2	—	925 ± 2312	5.6 ± 2.0
5	0.4	3	3.7	21.1	64,434	4.2	59.7	—	157 ± 435	3.8 ± 1.5
6	2.7	3	2.6	2.7	32,825	4.7	47.3	1401	134 ± 210	3.5 ± 2.3
7	1.4	4	6.1	41.4	100,719	3.9	70.0	—	542 ± 621	4.4 ± 1.8
8	2.2	4	1.8	4.5	9079	4.9	62.8	1662	70 ± 94	4.3 ± 1.8
9	1	1	2.8	36.7	13,553	6.7	61.5	1670	140 ± 266	3.8 ± 1.9
10	2.1	1	0.9	1.4	3557	10.8	73.1	—	140 ± 248	3.3 ± 1.1
11	2.2	1	0.9	4.8	4498	12.4	69.5	1956	299 ± 312	3.8 ± 1.1

*Note:* (%) = steepness of littoral zone area in depths 0–1 m, (i) = index value, (ha) = hectares. Turbidity sum is the monthly average turbidity of all seining occasions in the study sites. Heat sum 1 is the sum of average temperatures measured from three different depths at every seining occasion (0.3, 0.5 and 1.0 m) counted cumulatively. Heat sum 2 is cumulative temperature based on diurnal average data from automatic temperature loggers, covering the period 21.4.–18.8.2005 (unit = degree days).

## Appendix B The Total Catch Including All Subareas

**Table:**

Species, Adults	Sampling period, proportion of catch			
	May	June	July	August
Bleak (*Alburnus alburnus*)	2.93	4.89	17.73	32.41
Sand eels (Ammodytidae, *Ammodytes tobianus*/*Hyperoplus lanceolatus*)	25.69	33.46	22.17	7.56
Three spined stickleback (*Gasterosteus aculeatus*)	30.28	44.01	53.31	12.85
Black goby (*Gobius niger*)	0.07	—	0.02	—
Ruffe (*Gymnocephalus cernua*)	—	—	0.02	—
Straightnose pipefish (*Nerophis ophidion*)	0.05	0.08	0.07	0.48
Perch (*Perca fluviatilis*)	—	—	—	0.12
Minnow (*Phoxinus phoxinus*)	0.02	0.08	0.90	0.24
Flounder (*Platichtys flesus*)	0.58	1.42	0.20	—
Common goby (*Pomatochistus microps*)	23.43	8.21	4.33	30.49
Sand goby (*Pomatochistus minutus*)	13.29	3.72	0.72	14.89
Nine spined stickleback (*Pungitius pungitius*)	3.54	4.07	0.50	0.84
Roach (*Rutilus rutilus*)	0.02	—	—	—
Turbot (*Scopthalmus maximus*)	0.07	0.06	—	0.12
Broadnose pipefish (*Sygnathus typhle*)	—	0.02	0.02	—
Eelpout (*Zoarces viviparus*)	0.02	—	—	—
Total number of individuals	4297	5215	4438	833
Juveniles or larvae				
Bleak (*Alburnus alburnus*)	—	—	0.68	2.73
Garfish (*Belone belone*)	—	—	0.28	0.44
Herring or sprat (Clupeidae, *Clupea harengus*/*Sprattus sprattus*)	86.67	63.16	3.04	0.16
Whitefish (*Coregonus lavaretus*)	—	15.79	—	—
Three spined stickleback (*Gasterosteus aculeatus*)	13.33	10.53	1.97	3.44
Ruffe (*Gymnocephalus cernua*)	—	—	0.07	—
Straightnose pipefish (*Nerophis ophidion*)	—	—	—	0.05
Minnow (*Phoxinus phoxinus*)	—	—	0.05	—
Gobies (Pomatochistus sp.)	—	—	93.77	93.07
Nine spined stickleback (*Pungitius pungitius*)	—	10.53	0.12	0.05
Broadnose pipefish (*Sygnathus typhle*)	—	—	—	0.05
Total number of individuals	15	19	43,357	1832
Total number of seine hauls	99	132	66	33
Number of empty hauls	12	12	1	1
Surface area of hauls (m^2^)	12,375	16,500	8250	4125

*Note:* The table shows the species proportion of each month. Number of hauls, number of empty hauls and covered surface area given at the bottom of the table.

## Appendix C Comparative studies carried out in the northern Baltic Sea

**Table:**

Study	Area	Method	Number of species
Nellbring (1988)	Askö, Sweden	Beach seine and trap	18–19
Sundell (1994)	Hanko Peninsula, Finland	Beach seine	27
Rajasilta et al. (1999)	Archipelago Sea, Finland	Beach seine	15
Repečka, Stankus, and Lozys (2003)	Klaipëda‐Bûtingë, Lithuania	Beach seine	22
Lappalainen and Urho (2006)	Hanko Peninsula, Finland	Beach seine and detonation	18
Ustups, Uzars, and Müller‐Karulis (2007)	Pape and Jûrmalciems (Latvia)	Beach seine	31
Taal et al. (2017)	Gulf of Finland, Estonia	Beach seine	24
This study	Hanko Peninsula, Finland	Beach seine	20

*Note:* The majority of these studies included different habitats and were not restricted to the sandy beach only.
